# Antioxidant Activity of a Geopropolis from Northeast Brazil: Chemical Characterization and Likely Botanical Origin

**DOI:** 10.1155/2017/4024721

**Published:** 2017-10-31

**Authors:** Joselena M. Ferreira, Caroline C. Fernandes-Silva, Antonio Salatino, Dejair Message, Giuseppina Negri

**Affiliations:** ^1^Departamento de Ciências Animais, Universidade Federal Rural do Semi-Árido, Mossoró, RN, Brazil; ^2^Departamento de Botânica, Instituto de Biociências, Universidade de São Paulo, São Paulo, SP, Brazil; ^3^Universidade Federal de São Paulo, São Paulo, SP, Brazil

## Abstract

Geopropolis is a product containing wax, plant resin, and soil particles. It is elaborated by stingless bees of tribe Meliponini. Methanol extracts of sample of geopropolis produced by* Scaptotrigona postica* (“mandaguari”) in the state of Rio Grande do Norte (RN, northeast Brazil) were analyzed for the determination of standard parameters (total phenols, total flavonoids, and radical scavenging activity) and chemical characterization by HPLC-DAD-MS/MS analysis. The sample analyzed has high contents of total phenols and flavonoids, as well as high antioxidant activity. The constituents characterized were mainly flavonols, such as quercetin methyl ethers, and methoxychalcones. Such chemical profile is similar to the composition of a green propolis from the same area of RN, which is produced by Africanized* Apis mellifera*, using shoot apices of* Mimosa tenuiflora*, popularly known as “jurema-preta.” This finding provides evidence that “mandaguari” geopropolis and honeybee propolis have the same botanical origin in RN. The sharing of a plant resin source by phylogenetically distant bees (Apinae and Meliponinae) suggests that bee genetic factors play little role in the choice of plants for resin collection and that the availability of potential botanical sources plays a decisive role.

## 1. Introduction

Apidae and Meliponinae correspond, respectively, to a family and a subfamily of tropical bees. An important tribe in the group is Meliponini, whose members are known as stingless bees. There are over 300 species of Meliponini, distributed into several genera, including* Melipona* and* Trigona*. The former genus comprises about 50 exclusively neotropical species, while the former is a larger group, represented by over 130 pantropical species. They are valuable pollinators of either native or crop plants [1]. The hives of stingless bees are built as honeycombs for the larvae and as pots for honey and pollen keeping [2, 3].

Geopropolis is a product elaborated by stingless bees. It is similar to honeybee propolis, since both products contain plant resin and beeswax. They are distinct, however, because geopropolis contains variable content of soil material. As is the case with propolis, geopropolis is used to line the entrance of the hive and seal holes on the hive nest. Much have been published about chemistry of propolis, its botanical origin, and biological activity [4–6]. In comparison, considerably less has been done on geopropolis, in spite of the huge biological diversity of stingless bees [7, 8]. In Brazil, geopropolis has been used for the treatment of respiratory diseases and dermatoses [9]. Brazilian geopropolis revealed antioxidant, anticancer, anti-inflammatory, and antimicrobial activities [10], while Indonesian geopropolis has cytotoxic activity against several human cancer cell lines [11]. Most papers about chemistry of Brazilian geopropolis have dealt with* Melipona *species.* Melipona fasciculata* produces different types of geopropolis: carbohydrates, triterpenes, anacardic acid, alkylresorcinols, and sugar alcohols characterize a geopropolis from the northeast Brazil [7], while phenolic acids, gallo- and ellagitannins, are constituents of a geopropolis from the lowlands of the state of Maranhão (western northeast Brazil) [2, 3].* Melipona subnitida* from the state of Paraíba (northeast Brazil) produces geopropolis containing phenylpropanoids and flavonoids [12].* Melipona scutellaris* from the state of Bahia (southern northeast Brazil) produces distinct types of geopropolis, for example, a sample containing benzophenones as main constituents [13] and another one characterized by cinnamic acid esters and coumarins [8]. Flavonoid glycosides were detected in geopropolis of two Amazonian stingless bees,* Melipona interrupta *and* M. seminigra* [14], and phenolic compounds and terpenes were reported for a geopropolis of* M. orbignyi* from Mato Grosso do Sul (central-west Brazil) [15]. Much less has been published about geopropolis of other species of stingless bees. A geopropolis of* Trigona spinipes* contains triterpenes and magniferolic acid, among other constituents [9]. A geopropolis of* Scaptotrigona postica* from Maranhão has a peculiar chemical profile: in addition to caffeoyl-quinic acids, it contains flavone-*C*-glycosides (chiefly vicenin-2) and a pyrrolizidine alkaloid derived from retronecine [16].

Studies about botanical origin of geopropolis are scarce. Evidence has been raised that fruits of* Corymbia torelliana* (Myrtaceae) provide resin for the production of geopropolis by the Australian* Tetragonula carbonaria* (Meliponini), due to a great similarity in the methylated flavanone profiles of both materials [17].

To our knowledge, nothing has been published about the chemistry of geopropolis of* Scaptotrigona* from the Brazilian Semiarid, a region designated as the “drought polygon.” It has been shown recently that a dark green honeybee propolis from Rio Grande do Norte (RN) is produced with shoot apices of* Mimosa tenuiflora *(Willd.) Poir. (Leguminosae, Mimosoideae), a plant locally known as “jurema-preta” [18]. It is a hardy and abundant species in the caatinga biome, a typical dry forest of Brazilian northeast, characterized by spiny small trees and shrubs, cacti, and euphorbs. Most caatinga plants are devoid of leaves over the long dry season, but “jurema-preta” remains green all year long. In the same area, a geopropolis with dark green color is produced by bees of* Scaptotrigona postica *Latreille, 1807, popularly known as “mandaguari.” It is a highly adaptable and active pollinator stingless bee widespread in several parts of Brazil. Many meliponaries produce “mandaguari” pollen and propolis, the latter being reputed as having quality superior to the green propolis from southeast Brazil. The aim of the present study was to determine standard parameters (total phenolic substances, total flavonoids, and antioxidant activity) of* S. postica* geopropolis, characterize its constituents, and test the hypothesis that “jurema-preta” might be its resin source.

## 2. Material and Methods

### 2.1. Material

A geopropolis sample of* S. postica* was collected in January 2014 in the meliponary at UFERSA (Rural Federal University of the Semiarid), municipality of Mossoró, RN state (eastern northeast Brazil; 05°11′16′′S 37°20′38′′W). Nearly 3 g of material was collected from three boxes, by scraping the product accumulated between the hive and its cover. The geopropolis collected is dark green and somewhat sticky, with resinous smell and moldable texture. The sample was cleaned of impurities, placed inside plastic bags, and kept in freezer.

### 2.2. Preparation of Ethanol Extracts

The geopropolis sample was powdered using liquid nitrogen, mortar, and pestle. Powdered material (1 g) was treated with 150 mL of ethanol in Soxhlet for 6 h. The extract was filtered and kept overnight in a dark flask in freezer at −20°C. The cold extract was filtered again to eliminate wax excess.

### 2.3. Standard Chemical Parameters

An aliquot of 20 mL of the wax free extract (WFE) was evaporated to dryness under nitrogen flow and weighed for determination of total solids. Triplicates of 5 mL of WFE were used for determination of the contents of total phenolic substances and total flavonoids, the former by the method of Folin–Ciocalteu and the latter by the method of the aluminum chloride. The procedures were described elsewhere [19]. Reference compounds used were *p*-coumaric (total phenols) and quercetin (total flavonoids).

### 2.4. Antioxidant Activity

Triplicates of 5 mL of WFE were used for analysis of DPPH (2,2-diphenyl-1-picrylhydrazyl) free radical scavenging activity, according to Righi and collaborators [21], with modifications. Detailed procedure is available in a recent publication [18].

### 2.5. HPLC/DAD and HPLC-DAD-ESI-MS/MS Analyses

Aliquots of 1 mL of WFE were evaporated to dryness under nitrogen flow and the residue was dissolved in HPLC grade methanol to obtain solutions at 10 mg mL^−1^. The solutions were purified through 0.45 *μ*m filters. Aliquots (10 *μ*L) were analyzed by HPLC-DAD with a HPLC HP 1260 chromatograph (Agilent Technologies), using a Zorbax 5B-RP-18 column. Analysis was also carried out by HPLC-DAD-ESI-MS/MS with a DADSPD-M10AVP Shimadzu chromatograph equipped with degasser, two LC-20AC pumps, CTO-20A column oven, SIL 20AC autoinjector and SPD-20A, and a reverse phase column Phenomenex Gemini C-18, protected by a guard column. The chromatograph was coupled to an esquire 2000 Plus Bruker Daltonics spectrometer, equipped with a quadrupole ion trap mass analyzer. Details of the analysis procedures, such as gas flows, oven temperatures, solvent gradients, and databases for chemical characterization of constituents, are available in a recent publication [18]. Constituents' characterization was based on UV-DAD and MS data in the negative and positive ionization modes and comparison with previously published data [22–25].

## 3. Results and Discussion

### 3.1. Standardization and Antioxidant Activity

The contents of soluble solids, total phenolic compounds, and total flavonoids are shown in Table 1, in addition to the minimum acceptable values given by the Technical Regulation of Propolis Identity and Quality (TRPIQ) (standards for comparison of physical and chemical characteristics of propolis produced in Brazil) [20]. All parameters analyzed comply with TRPIQ standards. As apparent in the table, they are similar to values reported for RN green propolis derived from “jurema-preta” [18] and considerably higher than the corresponding parameters of the internationally marketed green propolis from southeast Brazil [19]. The EC_50_ antioxidant activity of the geopropolis sample is also shown in Table 1. As expected from the similarity between RN green geopropolis and “jurema-preta” propolis, the DPPH radical scavenging capacity of both bee products is also similar. The antioxidant activity of propolis has been attributed to its phenolic profile [26].

### 3.2. Chemical Characterization and Comparison with “Jurema-Preta” Propolis

Table 2 summarizes UV-DAD and MS data in the negative and positive ionization modes. Flavonoids were characterized by UV/vis bands at 345–360 nm (band I) and 255–280 nm (band II); chalcones were characterized by bands at 340–370 nm (band I) and flavanones by band at 280 nm [22]. The characterization of methoxylated flavonoids was based on fragments generated by loss of a methoxyl radical (^*∙*^CH3) and chalcones were characterized by fragments generated by Retro-Diels-Alder reaction [23–25]. Details about characterization of the compounds based on UV/vis, MS^−^, MS^+^, and sodium adducts ([M + Na]^+^) are available in the paper by Ferreira and collaborators [18].

**Figure 1:**
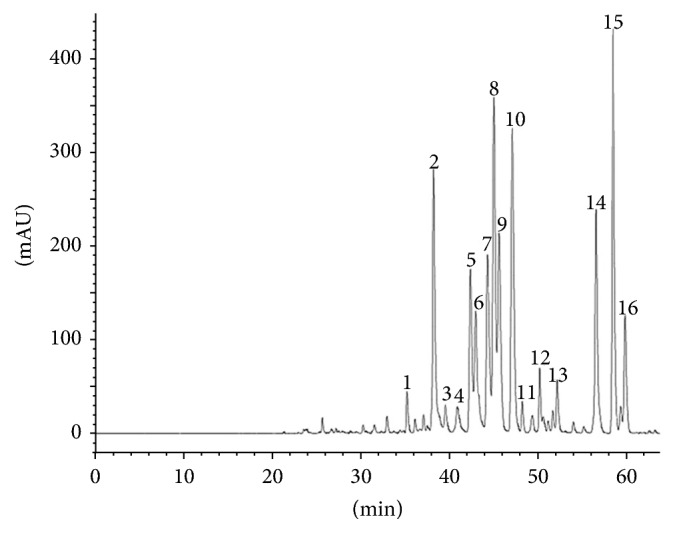
HPLC chromatogram of ethanol extracts of geopropolis of* Scaptotrigona postica* (“mandaguari”; Meliponini) from the meliponary at the University of Mossoró (state of Rio Grande do Norte, northeast Brazil), as characterized by HPLC-DAD. Digits of the chromatogram peaks correspond to the numbers seen in Table 2, used to indicate compounds characterized by HPLC-DAD-MS/MS.

Only phenolic compounds, virtually only flavonoids, were characterized as constituents of the analyzed extracts of geopropolis (Table 2). This observation is consistent with the exceptionally high flavonoid content shown in Table 1. Biological properties of propolis are often attributed to their flavonoid constituents. Compounds** 1**,** 4,** and** 11** are simple flavonol aglycones. Compounds** 2**,** 5**,** 7,** and** 9** are flavonol methyl ethers, compounds** 3**,** 6**,** 8**, and** 13**–**15** are chalcones, compound** 11** is a flavanone, and compounds** 12** and** 16** are flavones. Thus, the main constituents of the analyzed* S. postica* geopropolis are flavonols and chalcones.  Figure 1 depicts the HPLC-DAD chromatogram of the geopropolis ethanol extract. So far the origin of resin of propolis containing chalcones has been attributed to Leguminosae species, for example,* Dalbergia ecastophyllum*, the plant source of Brazilian red propolis [21, 27, 28]. The main plant sources of chalcones are species of Asteraceae, Leguminosae, and Moraceae [29]. The present report of chalcones as major constituents of both geopropolis of* S. postica* and green propolis of* Apis mellifera* from RN is coherent with the hypothesis that “jurema-preta” is one of their relevant botanical sources [18]. Data of Table 2 evidences the high similarity between chemical compositions of both products. Among the 16 constituents detected in the ethanol extract of the geopropolis, 10 were also detected as constituents of the green propolis from the same area (Table 2). “Jurema-preta” is abundant in the caatinga vegetation and its pollen is an important nutritional resource for the local bees.

### 3.3. Comparison with Other Geopropolis

The chemical profile seen in Table 2 is distinct from the composition reported for other Brazilian geopropolis. To our knowledge, no chalcones have so far been detected in geopropolis. A geopropolis of* Plebeia *aff.* flavocincta* from RN has been reported to contain high contents of phenols and flavonoids, but no detailed analysis was carried out for characterization of individual constituents [30]. The geopropolis analyzed in the present work is much distinct from a geopropolis produced by* Scaptotrigona postica* from Maranhão [16]. It is worth observing that stingless bee colonies of the same species may produce geopropolis much distinct from one another (as noted with* S. postica* from Maranhão and RN), while bees phylogenetically far apart (such as* Scaptotrigona* and* Apis*) may elaborate products with similar chemical composition. This observation leads to the conclusion that bee genetic factors play little role (if any) in the choice of plant sources for geopropolis and propolis production. The availability of potential resin sources is probably decisive in this regard. “Jurema-preta” plants fit well as propolis and geopropolis sources due to their abundance in the caatinga, even during the severe dry periods. In addition, they provide fresh tissues amenable to be chewed by the small and relatively delicate mandibles of the mouth apparatus of bees [31].

### 3.4. Perspectives

Favorable prospects regarding biological activities are suggested by the high content of flavonols and chalcones and the expressive antioxidant activity of the RN “mandaguari” geopropolis. In addition to many other biological activities, flavonols such as quercetin and isorhamnetin derivatives exhibit coronary heart disease prevention, as well as antioxidant, hepatoprotective, anti-inflammatory, and anticancer effects [32, 33]. Chalcones are flavonoids recognized as possessing many activities, such as antioxidant, anti-inflammatory, antibacterial, antifungal, cytotoxic, antitumoral, and chemopreventive activities [29, 34–36].

## 4. Conclusion

Most propolis in the commerce derives from honeybees. Some stingless bees, however, may produce propolis in amounts commercially feasible, as is the case of “jataí”* (Tetragonisca angustula)*, from the northeast of Brazil. Bees of* S. postica *(“mandaguari”) reveal promising prospects for becoming a relevant source of commercial geopropolis. The already reputed high quality of its geopropolis is consistent with results of the present paper, such as the high antioxidant activity and the exceptional predominance of flavonoids, including a high content of chalcones.

Taking into account the economic and social difficulties afflicting the population of northeast Brazil, including the population of RN, the implementation of actions stimulating the production of honeybee green propolis and “mandaguari” geopropolis, both products derived from “jurema-preta,” might afford benefits for the local economy, mainly during the harsher dry seasons.

## Figures and Tables

**Table 1:** Chemical parameters (g kg^−1^) and DPPH radical scavenging activity (*µ*g mL^−1^) of a sample of geopropolis of *Scaptotrigona postica* from Mossoró (state of Rio Grande do Norte, northeast Brazil), compared with mean values of “jurema-preta” propolis [18] and limit values of the Technical Regulation of Propolis Identity and Quality (TRPIQ) [20].

Sample	Soluble solids	Total phenols	Total flavonoids	EC_50_
Geopropolis	550	111.5 ± 5.4	98.5 ± 8.6	74.1 ± 0.5
“Jurema-preta” propolis	537	140	105	65
TRPIQ	Minimum 350	Minimum 50	Minimum 5	—
Quercetin	—	—	—	7.4 ± 0.1

**Table 2:** Distribution of constituents of ethanol extracts of geopropolis of *Scaptotrigona postica* (Meliponini) from the meliponary at the University of Mossoró (state of Rio Grande do Norte, northeast Brazil), as characterized by HPLC-DAD-ESI-MS/MS analysis. Data are also given about presence/absence of the constituents in propolis of *Apis mellifera* from the same area, derived from shoot apices of “jurema-preta” (*Mimosa tenuiflora*, Leguminosae), according to published data [18].

Compound number	Retention time	UV (nm)	MS^−^ (*m/z*, %)	MS^+^ (*m/z*, %)	Proposed characterization	References	Presence/absence in “jurema” propolis
1	35.2	260, 365	301 [M − H]^−^	303 [M + H]^+^	Quercetin	Comparison with standard	+
2	38.2	260, 365	315 [M − H]^−^	339 [M + Na]^+^, 317 [M + H]^+^, 302 (100)	Quercetin methyl ether	[19, 21]	+
3	39.6	270, 365	315 [M − H]^−^	339 [M + Na]^+^, 317 [M + H]^+^, 197 (100)	Trihydroxy-dimethoxy chalcone	[17–19, 22]	+
4	41.0	260, 360	285 [M − H]^−^	287 [M + H]^+^	Kaempferol	Comparison with standard	−
5	42.4	260, 360	299 [M + H]^−^	323 [M + Na]^+^, 301 [M + H]^+^, 286 (100)	Kaempferol methyl ether	[19, 21]	+
6	43.0	250, 270sh, 365	329 [M − H]^−^	353 [M + Na]^+^, 331 [M + H]^+^, 316 (100)	Dihydroxy-trimethoxy chalcone	[19, 21]	+
7	44.4	255, 355	313 [M − H]^−^	337 [M + Na]^+^, 315 [M + H]^+^, 300 (60), 282 (100)	Kaempferol dimethyl ether	[19, 21]	+
8	45.1	370	315 [M − H]^−^	317 [M + H]^−^	Uncharacterized chalcone	—	−
9	45.6	255, 355	329 [M − H]^−^	353 [M + Na]^+^, 331 [M + H]^+^	Quercetin dimethyl ether	[19, 21]	+
10	47.1	255, 355	327 [M − H]^−^	329 [M + H]^+^	Uncharacterized flavonol	—	−
11	48.3	260, 360	271 [M − H]^−^	273 [M + H]^+^	Trihydroxy flavonol	[19, 21]	−
12	50.2	270, 340	329 [M − H]^−^	331 [M + H]^+^	Trihydroxy-dimethoxy flavone	[19, 21]	−
13	52.2	370	269 [M − H]^−^	271 [M + H]^+^, 161 (100), 137 (70)	Methoxy-dihydroxy chalcone	[17–19, 22]	+
14	56.6	370	299 [M − H]^−^	301 [M + H]^+^, 167 (100)	Dimethoxy-dihydroxy chalcone	[17–19, 22]	+
15	58.5	370	313 [M − H]^−^	315 [M + H]^+^	Hydroxy-trimethoxy chalcone	[17–19, 22]	−
16	59.9	270, 340	343 [M − H]^−^	367 [M + Na]^+^, 345 [M + H]^+^	Dihydroxy-trimethoxy flavone	[17–19, 22]	+
