# Corrigendum to “Antioxidant Activity of a Geopropolis from Northeast Brazil: Chemical Characterization and Likely Botanical Origin”

**DOI:** 10.1155/2018/7084284

**Published:** 2018-04-19

**Authors:** Joselena M. Ferreira, Caroline C. Fernandes-Silva, Antonio Salatino, Dejair Message, Giuseppina Negri

**Affiliations:** ^1^Departamento de Ciências Animais, Universidade Federal Rural do Semi-Árido, Mossoró, RN, Brazil; ^2^Departamento de Botânica, Instituto de Biociências, Universidade de São Paulo, São Paulo, SP, Brazil; ^3^Universidade Federal de São Paulo, São Paulo, SP, Brazil

In the article titled “Antioxidant Activity of a Geopropolis from Northeast Brazil: Chemical Characterization and Likely Botanical Origin” [1], the scientific name of the stingless bee was given as* Scaptotrigona postica*. However, it must be corrected to* Scaptotrigona *aff.* depillis*. The change has been suggested by Dr. Airton Carvalho, expert on taxonomy of stingless bees. According to Dr. Carvalho, so far no formal description and publication of the bee has been done. Voucher specimens have been deposited in the collection ASA (Abelhas do Semiárido—Bees from the Semiarid).
